# Transient jejuno-jejunal intussusception in an anabolic steroid user—A case report

**DOI:** 10.1016/j.ijscr.2020.04.010

**Published:** 2020-05-08

**Authors:** Marie Shella De Robles, Robert Sean O’Neill, Christopher J. Young

**Affiliations:** a Department of Colorectal Surgery, Royal Prince Alfred Hospital, Sydney, New South Wales, Australia; b School of Medicine, University of Notre Dame, Darlinghurst, Australia; c The University of Sydney, Discipline of Surgery, Sydney, New South Wales, Australia

**Keywords:** Case report, Intussusception, Intestinal obstruction, Jejunum, Steroids

## Abstract

**Introduction:**

Adult intussusception (AI) is both a challenging and rare diagnosis, with predisposing factors including malignancy, surgery and infection to name a few. Transient jejunal intussusception is a subset of AI which is usually diagnosed radiologically, with diagnostic laparoscopy utilised to determine whether a malignant cause is identifiable and subsequently treatable.

**Presenting case:**

We present the case of a previously healthy 36-year-old male diagnosed with transient jejunal intussusception on computed tomography after presenting with abdominal pain. Blood tests on admission were normal apart from polycythaemia. His only significant history was that of chronic anabolic steroid use. He had a subsequent normal gastroscopy and colonoscopy with diagnostic laparoscopy demonstrating thickening of the small bowel. Histopathological analysis of the intraoperative specimen was normal. The patient improved and was discharged with no further complications.

**Conclusion:**

This case highlights the potential association between anabolic steroid use resulting in polycythaemia, and AI or transient jejunal intussusception, along with further validating a conservative approach in the management of AI in patients deemed to be low risk of malignancy on pre-operative evaluation.

## Highlights

• Adult intussusception is a rare cause of abdominal pain with the majority of presentations being localised to the small bowel.
• Transient jejunal intussusception is usually diagnosed on CT with diagnostic laparoscopy performed to confirm resolution and exclude malignant causes.
• Polycythaemia was noted on the patient’s blood results which has been previously associated with anabolic steroid use and has been associated with intussusception in the literature.
• Laparoscopic and endoscopic examination demonstrated nil malignant or structural cause for intussusception in the presented patient.
• Anabolic steroid use may play a role in the development of intussusception in the adult population.

## 1 Introduction

Adult intussusception (AI) is a challenging diagnosis. Reported frequently in children, intussusception is a rare presentation in adults, with an incidence of 2–3 per 1000000 and a predominance in females [1]. In addition to this, AI accounts for only 1–5% of bowel obstructions with a pathologic lead point identifiable in up to 90 % of cases [2,3]. Most AIs arise from the small bowel, with the majority of lesions being benign with a rate of 50–75% in published series [[4], [5], [6]]. The predominant cause of AI is neoplastic of which both malignant and benign neoplasms have been implicated. Although malignancy is implicated in a large majority of AI, aetiologies of AI include surgery-related AI, idiopathic and infection among others [7].

Transient jejunal intussusception is a rare pathological condition and is usually a diagnosis of exclusion, with risk factors including Crohn’s disease, celiac disease, inflammation and adhesions [[8], [9], [10], [11], [12], [13]]. Computed tomography (CT) scan remains the gold standard for diagnosis while diagnostic laparoscopy is an essential tool in distinguishing transient from persistent intussusception by excluding a pathological process such as a tumour. The current report presents the case of an adult male who was diagnosed with jejuno-jejunal intussusception on abdominal CT which had subsequently resolved on diagnostic laparoscopy. His medical history was complicated by chronic anabolic steroid use. The work has been reported in line with the SCARE criteria [14]. The informed consent was obtained from the patient for publication of this case report.

## 2 Case presentation

A 36-year-old male presented to the emergency department of a metropolitan hospital complaining of abdominal pain, described as intermittent and crampy. He denied previous episodes of similar pain in the past. He denied any previous medical history with nil previous abdominal surgery reported. On further history he admitted to the chronic use of oxandrolone, an androgen and anabolic steroid. On examination, he was haemodynamically stable, with periumbilical tenderness detected on abdominal palpation. Examination was otherwise remarkable.

**Fig. 1:**
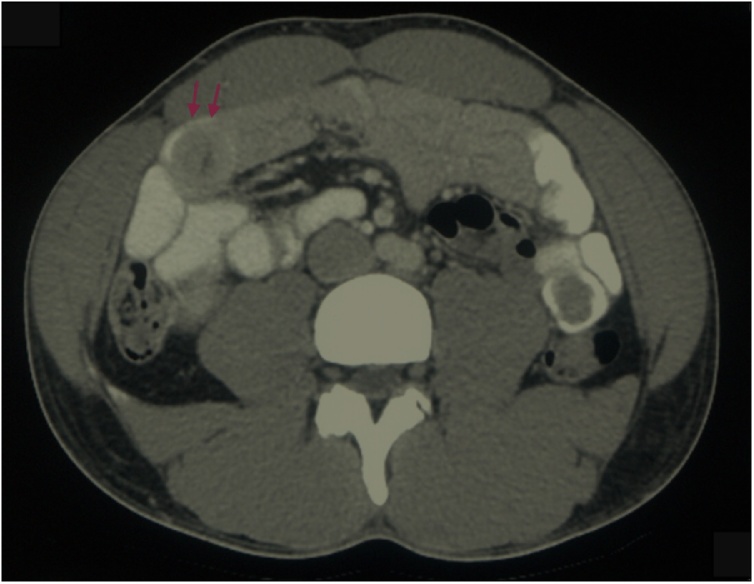
CT abdomen axial view demonstrating mild diffuse small bowel wall thickening, with two separate areas of apparent short segment jejunal intussusception without evidence of obstruction, and the characteristic ‘target sign’ indicative of intussusception (red arrows).

**Fig. 2:**
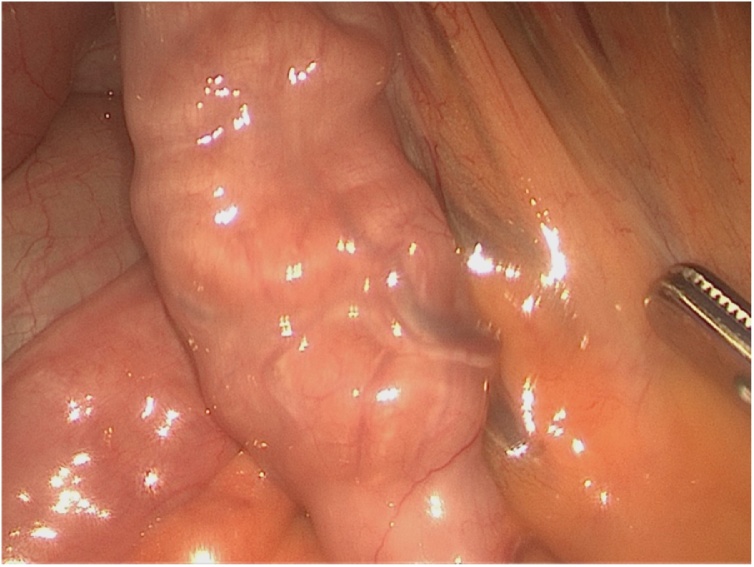
Laparoscopic findings demonstrating thickened small bowel loop with nil evidence of intussusception or bowel obstruction.

Blood samples were obtained from the patient which revealed an elevated haemoglobin at 164 g/L and haematocrit of 50 %. CT scan of the patient’s abdomen demonstrated mild diffuse small bowel wall thickening, with two separate areas of apparent short segment jejunal intussusception without evidence of obstruction (Fig. 1). After review of the abdominal CT, the patient immediaetely proceeded to gastroscopy and colonoscopy on the day of presentation which were both unremarkable. A diagnostic laparoscopy on the day of presentation was subsequently performed, which was negative for intussusception or potential pathological lead point. Small bowel thickening was noted, and a biopsy was obtained for histopathological assessment (Fig. 2).

Histopathological assessment revealed normal villous architecture and inflammatory cells within normal limits. No evidence of dysplasia or malignancy was reported.

With the apparent resolution of the small bowl intussusception and abdominal pain, the patient was discharged home. He was advised on the cessation of regular anabolic steroid use and on routine follow-up, the patient reported significant improvement in abdominal pain with no further presenting episodes.

## 3 Discussion

Intussusception if characterised as the invagination of a proximal segment (intussusceptum) into an adjacent distal segment of bowel (intussuscipiens). This presented case highlights the clinical phenomenon of transient jejunal intussusception in a previously healthy male. Transient intussusceptions have not been well reported in the literature, and its pathophysiology is poorly understood. Steroid-induced polycythemia can cause hyperviscosity and consequent mechanical vascular obstruction. These physiologic changes put patients with a history of chronic anabolic steroid use at an increased risk for ischemic events [15]. It has also been reported that androgens lead to a procoagulant state by potentiating platelet aggregation, increasing the production of thromboxane A2 or decreasing prostaglandin production [16]. This pro-coagulant state and polycythemia can potentially lead to increased risk for mesenteric ischemia and its consequent bowel wall thickening. The later can act as a focal area of traction or “lead point” that draws the proximal bowel within the peristalsing distal bowel. Similar to our case, three similar case reports have demonstrated that polycythemia can lead to bowel ischemia with resulting bowel thickening that they also postulated acted as a lead point for intussusception [15,[17], [18], [19], [20]]. The authors acknowledge that although the presented patient’s haemoglobin was only mildly elevated, his lack of comorbid conditions, normal preoperative biochemistry, and normal intraoperative biopsy, further strengthen the argument that in a healthy male, anabolic steroid induced polycythaemia is a potential cause of adult intussusception.

The clinical presentation of intussusception is variable but generally marked by abdominal pain and signs of bowel obstruction. Some patients may also present with nausea, vomiting, bleeding, changes in bowel movements, constipation or bloating [[4], [5], [6]]. Persistence of symptoms can occur due to continued peristaltic contractions of the intussuscepted bowel segment against the obstruction [11]. With continued invagination resulting in oedema, eventually, the vascular flow to the bowel becomes compromised, resulting in ischemia to the affected segment that can result in necrosis and perforation.

Given the non-specific clinical presentation and the vast differential, choice of imaging modality is critical to arriving at a timely diagnosis. CT scan remains the gold standard to obtain a diagnosis and elucidate a possible aetiology, with a reported sensitivity and specificity of 80 % and 100 % respectively [6,21]. The characteristic features would demonstrate a sausage-shaped soft tissue mass, or the classic ‘target’ or ‘doughnut’ sign showing concentric rings due to duplication of small bowel layers. The presented patient had an abdominal CT on presentation which demonstrated diffuse small bowel wall thickening with areas of apparent jejunal intussusception, along with the presence of a ‘target sign’, which is highly suggestive of intussusception. CT scan also provides additional information including the type and location of intussusception, length, and diameter, possible lead point, mesenteric vasculature, the possibility of strangulation and obstruction, or signs suggestive of malignancy [22]. In some patients with non-specific clinical symptoms and non-pathognomonic radiologic findings, diagnosis can only be made through a laparoscopy. In cases where intussusception is associated with a pathologic lead point and signs of bowel obstruction or GI bleeding, an exploratory laparotomy should be performed, as was the case with the presented patient.

In general, intraoperative management of AI is mostly done by en bloc resection without attempts at reduction if there are any doubts regarding a benign aetiology preoperatively on history, examination and radiological assessment (acute bowel obstruction, mesentery jeopardization or signs suggestive of malignancy) [23]. While surgery is often employed when intussusception is associated with an underlying pathological lead point such as a benign polyp, lipoma, Meckel diverticulum, malignancy, arteriovenous malformations or localised inflammation secondary to infection or chronic inflammation, its use in transient cases is still unclear with there being no formal guidelines for surgical exploration for idiopathic, transient small bowel intussusception [[24], [25], [26]]. Previous literature has demonstrated that larger small bowel intussusceptions are more likely to require surgical intervention, while those measuring less than 3.5 cm in length are more likely self-limiting and will resolve with conservative measures and follow-up imaging studies to monitor the resolution status of intussusception [[27], [28], [29]]. In addition to this, although surgery remains the mainstay of management in adult intussusception, enteric intussusception can be managed by reduction followed by resection, while colonic intussusception should be resected en bloc [30]. The rationale behind *en-bloc* resection in intussusception is based on a theoretical risk of venous embolization of potential malignanct cells on manipulation of the affected segment of bowel, and the risk of bowel perforation resulting in peritoneal seeding of malignant cells. There is a definitive lack of evidence to support this theory. A recent meta-analysis reported small bowel malignancy being the cause of intussusception in 22.5 % and 36.9 % of enteric and ileocolic intussusceptions respectively, thus supporting the approach for laparoscopic reduction and subsequent resection [30]. The authors argue that the presented patient’s lack of features suggestive of malignancy on clinical history, biochemistry and radiology, and subsequent apparent resolution of intussusception on diagnostic laparoscopy favour a conservative approach. This is further supported by a normal intra-operative biopsy of the affected portion of small bowel. Further research is needed to shed light on how long conservative management should be considered until escalation to surgical management is indicated in patients deemed low risk on history, clinical examination, biochemistry and radiological assessment [[4], [5], [6]].

## 4 Conclusion

Transient jejunal intussusception, a rare form of AI, is usually idiopathic in nature and diagnosed on radiological analysis. This report highlights a potential relation of transient jejunal intussusception to chronic anabolic steroid use. In addition to this, this report provides further evidence for a conservative approach in the management of AI in patients with clinical and radiological features which are contrary to the diagnosis of malignancy.

## Declaration of Competing Interest

The authors declare no conflicts of interest in the production of this case report.

## Funding

This case report had no sponsors.

## Ethical approval

This case report is exempt from ethical approval by our institution.

## Consent

Informed consent was obtained from the patient for publication of this case report.

## Author contribution

Dr Marie Shella De Robles and Dr Robert O’Neill were involved in the writing of the presented case report. Associate Professor Christopher Young and Dr Marie Shella De Robles were involved in the clinical care of the patient.

## Registration of research studies

This is not a ‘first in humans’ report, so it is not in need of registration.

## Guarantor

Associate Professor Christopher Young.

## Provenance and peer review

Not commissioned, externally peer-reviewed.
